# Practicing traditional Chinese medicine in the COVID-19 pandemic in Switzerland – an exploratory study

**DOI:** 10.1186/s12906-022-03715-w

**Published:** 2022-09-15

**Authors:** Angélique Bourqui, Pierre-Yves Rodondi, Emna El May, Julie Dubois

**Affiliations:** 1 Institute of Family Medicine, Faculty of Science and Medicine, University of Fribourg, Fribourg, Switzerland; 2 Population Health Laboratory (#PopHealthLab), Faculty of Science and Medicine, University of Fribourg, Fribourg, Switzerland

**Keywords:** TCM, Switzerland, Therapist, Physician, COVID-19

## Abstract

**Background:**

To curb the spread of the first wave of the COVID-19 pandemic, the Swiss government declared a state of health emergency and ordered a legal restriction concerning the opening of healthcare institutions. In this study, we aimed to assess the proportion of traditional Chinese medicine (TCM) physicians and therapists who consulted patients regarding COVID-19 during the first wave of the pandemic in 2020 in Switzerland, as well as the extent to which COVID-19 affected their practices during the same period.

**Methods:**

A retrospective study was performed by using a questionnaire from January to April 2021 among a random sample of TCM physicians and therapists based in Switzerland. The survey included questions on demographic characteristics, opening status of practices, channels of communication used for the medical encounter, and experience in managing the prevention, acute, and recovery stages of COVID-19 infection.

**Results:**

Among the 320 participants, 76% consulted a patient regarding COVID-19 at least once. Overall, physicians and therapists consulted more patients during recovery (76.3%) and prevention (67.8%) than during the acute stage (19.8%) of the disease. Acupuncture was the most frequently used technique among TCM therapists and physicians consulting for prevention (80.4%) and recovery (92.5%), whereas Chinese pharmacopeia was the most used technique among those consulting for the acute stage (59.3%). Of those who closed their practices from March to April 2020 but kept consulting, telephone (30.4%) and home visits (29.9%) were the two principal methods of consultation.

**Conclusions:**

The restriction concerning the opening of practices induced a loss of the health workforce, especially among TCM therapists. Nonetheless, TCM therapists and physicians consulted patients regarding COVID-19, especially during the recovery stage. As there is a demand for the use of TCM in the context of COVID-19, it raises the need for a better consideration of TCM in the Swiss health care system.

## Introduction

### Background

On February 25, 2020, the first Swiss case of COVID-19 was reported in Ticino, the Italian-speaking part of the country [1]. Since then, according to the Federal Office of Public Health, as of April 19, 2022, a total of 3,568,616 positive cases and 13,647 deaths have been reported in Switzerland, which has a population of 8.5 million [2]. As of March 16, 2020, the Swiss government declared a state of health emergency throughout the country, assigned the population to a semi-lockdown, and ordered the closure of almost all public establishments, apart from shops that provided basic necessities, in order to curb the spread of the infection [3]. From March 16 to April 27, 2020, a legal restriction concerning the opening of healthcare institutions was applied. Only “hospitals, clinics and medical practices, as well as practices and facilities operated by healthcare specialists under federal and cantonal law” [3] could remain open. Healthcare specialists under federal law included, among others, pharmacists, dentists, nurses and physiotherapists [4]. Most complementary and alternative medicine (CAM) professions are not recognized as health professions at the federal level and are regulated by cantonal laws, including traditional Chinese medicine (TCM). Thus, depending on their location, some CAM practices had to close, whereas others could remain open. During this period, however, all these institutions were prohibited from carrying out non-urgent examinations and therapies [3]. Thus, it is likely that most CAM practices had to close. This period pushed health practitioners to carry out remote consultations using digital settings such as videoconference [5–9]. However, the number of consultations decreased and induced financial losses for 99% of the sample of GPs interviewed [10]. After 6 weeks of semi-lockdown, a progressive reopening phase began. From April 28 to May 10, 2020, all healthcare practices were allowed to “resume normal operation and carry out all medical procedures, including non-urgent procedures” [11]. “Businesses offering personal services involving physical contact” could also reopen [11], which included all CAM practices. From May 11, 2020, the state of emergency was lifted, resulting in the reopening of schools, other businesses, and entertainment venues. On May 11, 2020, the state of emergency was lifted [12].

According to the Swiss health survey, one in four Swiss citizens used CAM in 2012 [13]. Among them, 6.8% reported using TCM, making it the third most popular CAM [13]. In Switzerland, TCM can be provided by trained physicians or therapists. To practice TCM, physicians have to hold a specialist title delivered by the Swiss Institute of Medical Education (ISFM) completed with a complementary training in “Acupuncture and Chinese pharmacopeia – TCM” [14]. Mandatory basic healthcare insurance will cover TCM treatments provided by licensed TCM physicians [15]. Regarding TCM therapists, there is no official training program [8]. To evaluate the qualification of TCM therapists, private organizations, mainly the Swiss Foundation for Complementary Medicine (ASCA) and the Empirical Medicine Register (RME), provide accreditation to TCM therapists by certifying their quality of care and their training based on a minimum number of hours of professional training in the area [15, 16]. Private supplemental insurances will cover TCM provided by therapists if they are registered in at least one of these private organizations.

In China, since the third version of the national guidelines for the prevention and treatment of COVID-19, the National Health Commission has incorporated TCM into their recommendations according to the stage of the disease (early, intermediate, severe, recovery) [17, 18]. According to the National Health Commission of the People’s Republic of China, it is estimated that 85% of patients diagnosed with COVID-19 in China have received TCM [18]. Additionally, numerous studies related to the use of TCM in the management of COVID-19 have already been published [19–21]. Some studies have shown, for example, a trend toward mortality reduction by adding TCM to conventional medicine [22]. Despite heterogeneity in the quality of RCTs conducted so far, TCM might be an interesting complementary approach in the management of COVID-19 patients.

Although a fair amount of studies on the management of COVID-19 with TCM, only a few assessed the use of CAM in relation to COVID-19 in Europe [23] or the impact of the legal restrictions on providers [24, 25], and to our knowledge, none focused on TCM specifically. Therefore, and given that legal restrictions in Switzerland may have affected TCM providers differently, we aimed to assess the proportion of TCM physicians and TCM therapists who consulted patients with respect to COVID-19 in Switzerland from March to September 2020, as well as the extent to which COVID-19 affected their practices during the same period.

## Material and Methods

### Study design

This retrospective cross-sectional study was carried out by means of a questionnaire among registered TCM physicians and TCM therapists based in Switzerland.

### Setting and participants

Survey participants were recruited by using the ASCA database for TCM therapists and the SASIS SA database for TCM physicians; SASIS SA is a Swiss register of service providers.

TCM therapists registered in the ASCA database were informed by ASCA on our behalf about the research project through an email that contained a letter of information about the research and a link to the online questionnaire. TCM physicians were contacted directly by the Institute of Family Medicine of the University of Fribourg through the database of SASIS. They were informed by postal mail, as not all TCM physicians had an email address registered in this database. The mail included an information letter, a questionnaire in paper form, and a prepaid envelope. They had the possibility to fill out the questionnaire in paper form or online by means of a QR code or by copying a URL link into their computer’s internet browser; both were available in the information letter. One reminder was sent after 3 weeks through the same channels to encourage participants to answer. TCM physicians and therapists were contacted in the main language spoken in their canton. If they wished, they could request a version of the questionnaire in another language (French, German, or English). Data collection occurred from January 15 to April 15, 2021. Inclusion criteria were that TCM therapists had to be registered in ASCA and TCM physicians had to be certified in acupuncture and Chinese pharmacopeia delivered by the ISFM. Moreover, therapists and physicians had to practice in Switzerland. Exclusion criteria were being retired or no longer consulting and/or not understanding French, German, or English. To be selected for the analysis, participants had to complete at least three of the four sections of the questionnaire.

For insight into the experience lived by TCM physicians and therapists, we conducted four preliminary interviews with them and three with pharmacists about producing and dispensing herbs. These preliminary interviews allowed us to design the questionnaire. It was then reviewed and tested for face and content validity by a TCM physician and a TCM therapist, as well as by colleagues external to this research project. The goal was to determine how surveyed persons interpreted the intent and meaning of survey questions. Modifications were then introduced to enhance participant comprehension of the questions. The questionnaire was anonymous. The Cantonal Commission for the Ethics of Human Research of Vaud (CER-VD) (2020-02813) approved the study.

### Variables

The primary outcomes were the proportion of TCM therapists and physicians who had consulted patients with respect to COVID-19 from March to September 2020, as well as the prevalence of consultations related to the stage of the disease (prevention, acute, recovery) and its associated factors. The secondary outcomes were the proportion of practices that closed during that same period, as well as the potential channels of communication used by therapists and physicians to perform their consultations.

To obtain this information, we developed a 38-item questionnaire divided into four sections, using Limesurvey software (version 3.17). The first two sections (seven items) explored sociodemographic data. The third section (five items) assessed the influence of the pandemic on clinical activity of TCM physicians and therapists by focusing specifically on three periods (March 16 to April 27, April 28 to May 10, May 11 to September 30, 2020). As described in the introduction, these three periods correspond to the three phases of legal restrictions put in place by the Swiss government in order to curb the spread of the infections during the first wave of Covid-19 in the country. We chose to restrict the period of interest to the first wave of COVID-19 and to give a delimiting date of September 30, as the restrictions differed between the different waves of COVID-19. This third section of the questionnaire covered aspects such as closure of practices and alternative ways to consult. In the fourth section (26 items), we investigated the experience of TCM physicians and therapists in managing patients with COVID-19-related symptoms based on three stages of the disease (prevention, acute, recovery). For each stage of COVID-19, we asked similar questions relative to the opinion of physicians and therapists on the role of TCM, their experience in managing COVID-19, and the techniques used. If Chinese pharmacopeia was used, we asked physicians and therapists to specify which herbs were prescribed.

### Statistical method

Statistical analyses were performed with R statistical software (version 1.2.5019). Standard descriptive analyses were performed for continuous variables (mean and standard deviation) and for categorical variables (N and percentages). A t-test was used to examine whether there was a significant relationship between profession and age. Pearson’s chi-square tests were used to examine whether there was a significant association between profession (therapist vs physician) and (i) gender; (ii) TCM techniques used; (iii) practice closing periods; (iv) role of TCM for COVID-19 prevention, treatment, and recovery; and (v) patients’ consultations for COVID-19 prevention, treatment, and recovery. Fisher’s tests were chosen over Pearson’s chi-square tests when the expected frequency of each cell was lower than five. We also calculated odds ratio for each Pearson’s chi-square test to have a measure of effect size. Additional loglinear analyses were conducted to evaluate whether age and/or sex influenced the relationship between the profession and practice closing period. A technical problem occurred in the design of the questionnaire that allowed the practitioners who reported closing during a period to indicate that they were still seeing patients at their practices during the same period. Therefore, analyses were done by excluding the answers of these professionals. The answer options “no,” “rather no,” “yes,” and “rather yes” were recoded into two groups: “yes” = (“yes, rather yes”) and “no” = (“no, rather no”).

## Results

### Participants

**Fig. 1:**
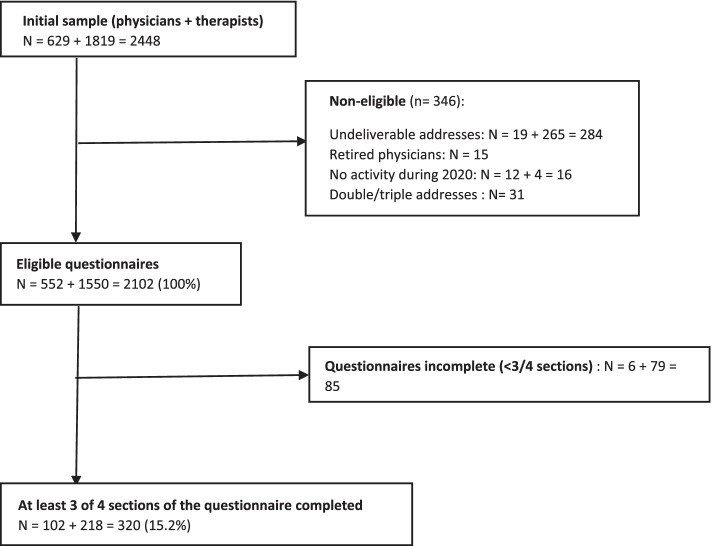
Flowchart

Among the 2102 eligible participants initially contacted by email or by post, 320 completed at least three of the four sections (response rate 15%). Among those, 102 were physicians and 218 were therapists, which corresponds to 18 and 14% of the initial sample of each population, respectively. A flowchart is shown in Fig. 1.

### Descriptive data

Among the participants, 31.9% were physicians and 68.1% therapists. Most respondents were women, with significantly more women among therapists than among physicians. The mean age of respondents was 52.3 years. On average, therapists appeared to be significantly younger than physicians (*p* < 0.001). Acupuncture was the most generally used technique among physicians and therapists. The second most used technique varied between physicians and therapists: moxibustion for TCM therapists and Chinese pharmacopeia for TCM physicians. Among the different techniques used, only tuina, moxibustion, and acupressure were significantly more used by therapists than physicians. Detailed sociodemographic data are reported in Table 1.

**Table 1:** Socio-demographic characteristics and techniques used by participants

Characteristic	Total (%)	TCM physicians (%)	TCM therapists (%)	*p*-value
Number of participants	320 (100)	102 (31.9)	218 (68.1)	
Gender				< 0.001^1^
Female	211 (65.9)	43 (42.2)	168 (77.1)	
Male	108 (33.8)	59 (57.8)	49 (22.5)	
Other	1 (0.3)	–	1 (0.5)	
Age group				< 0.001^1^
26-45 years	86 (26.9)	9 (8.8)	77 (35.3)	
46-64 years	186 (58.1)	63 (61.8)	123 (56.4)	
65+ years	41 (12.8)	28 (27.5)	13 (6)	
No answer	7 (2.2)	2 (2)	5 (2.3)	
Mean age (standard deviation)	52.3 (10.6)	58.1 (10.3)	49.5 (9.5)	< 0.001^2^
Age range	[26-87]	[34-87]	[26-76]	
TCM techniques generally used by participants				
Acupuncture	311 (97.2)	99 (97.1)	212 (97.3)	0.924^1^
Moxibustion	206 (64.4)	32 (31.4)	174 (79.8)	< 0.001^1^
Tuina	132 (41.3)	13 (12.8)	119 (54.6)	< 0.001^1^
Chinese pharmacopeia	130 (40.6)	35 (34.3)	95 (43.6)	0.116^1^
Acupressure	89 (27.8)	21 (20.6)	68 (31.2)	0.048^1^
Other	99 (39.7)	28 (27.5)	71(32.6)	0.356^1^

^1^ Pearson chi^2^

^2^ T-test

### Attitudes toward TCM for the prevention, acute, and recovery stages of COVID-19

**Fig. 2:**
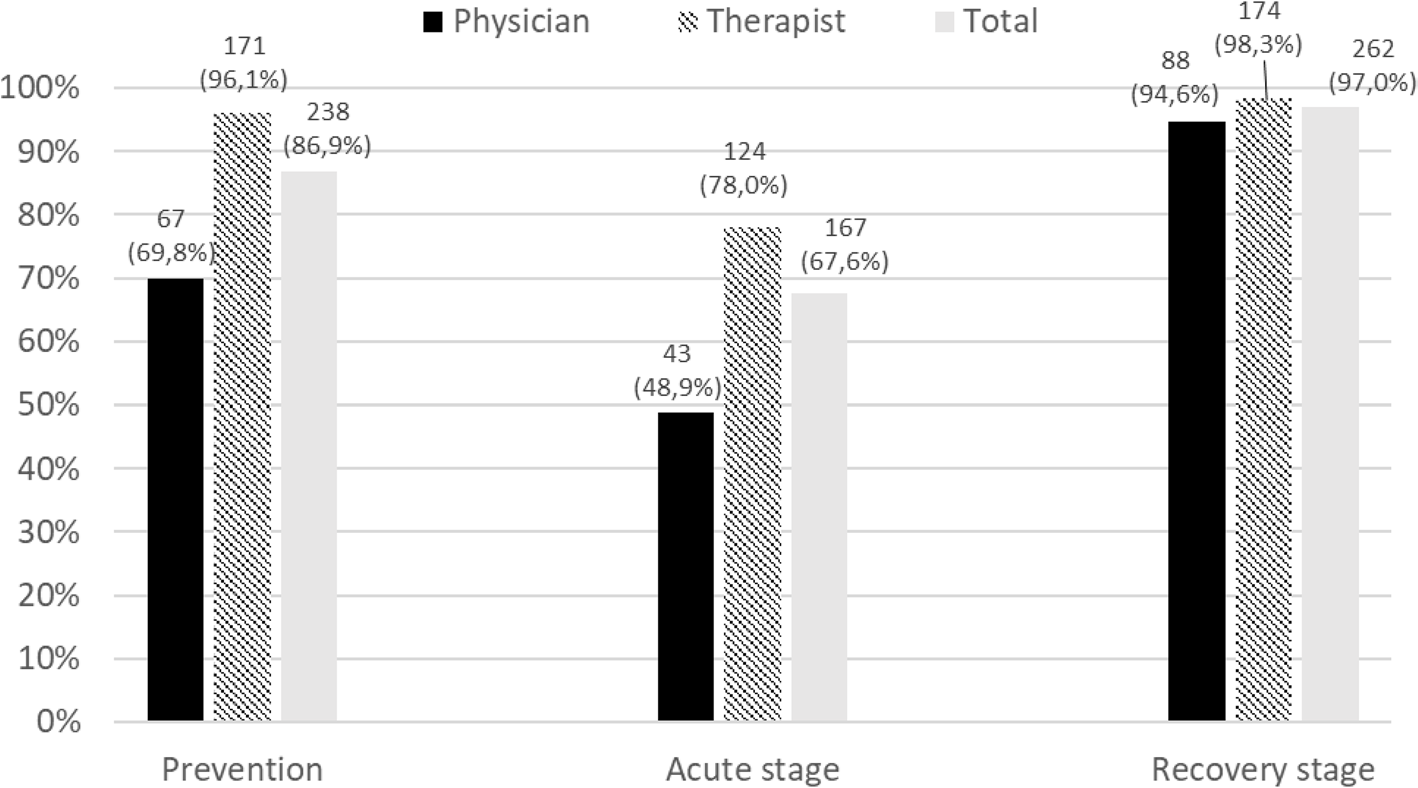
Number and percentage of TCM physicians and therapists who thought that TCM had a role to play in the management of COVID-19 according to the different stages of COVID-19 (prevention, acute, recovery)

Regarding the role played by TCM in the management of COVID-19 at different stages of the disease, opinions regarding its usefulness varied significantly between therapists and physicians in the prevention and acute stages (*p* < 0.001). Indeed, a higher proportion of therapists than physicians thought that TCM had a role to play in these stages. Nevertheless, almost all respondents agreed that TCM had a role to play in the recovery stage of COVID-19 (see Fig. 2).

### Outcome data

We explored the proportion of practices that closed during each of the three periods between March and September 2020, as well as the adaptations made by TCM therapists and physicians to continue consulting despite the pandemic.

Overall, the first period, from March 16 to April 27, recorded the highest percentage of closed practices. During this period, therapists closed their practices significantly more often than physicians did: 73.5% vs 35.6%, respectively (*p* < 0.01). The proportion of closed practices is presented in Table 2.

**Table 2:** Proportion of TCM physicians’ and therapists’ practices closed during each of the three periods in Switzerland

	Closed TCM physicians’ practices N (%)	Closed TCM therapists’ practices N (%)	Total of closed practices N (%)	***p***-value
March 16, 2020 to April 27, 2020 *N =* 316	36 (35.6)	158 (73.5)	194 (61.4)	p < 0.01
April 28, 2020 to May 11, 2020 *N =* 317	11 (10.8)	25 (11.5)	36 (11.3)	*p* = 0.86
May 11, 2020 to September 30, 2020 *N =* 318	3 (2.9)	9 (4.1)	12 (3.8)	*p* = 0.76

From April 28 to May 11, a notable reduction in the number of closed TCM physicians’ and therapists’ practices was observed compared with that in the first period. During the last period, almost no practice was closed. Loglinear analysis showed that neither age nor gender played a role in the closure of practices during this period.

Among TCM physicians and therapists who closed their practices from March 16 to April 27 (*N =* 194, 61.4%), 25% of TCM physicians (*N =* 9) and 47.5% of TCM therapists (*N =* 75) did not consult at all. For those who did consult while being closed over this period (*N =* 110, 56.7%), two main methods of consultation were used: telephone (*N =* 59, 30.4%) and home visits (*N =* 58, 29.9%). Among TCM physicians and therapists whose practices remained open in that same period (*N =* 122, 38.6%), 90.2% consulted at their practice, 49.2% did home visits, and 33.6% consulted by phone. Videoconference was used by 13% of those who closed the practice and by 14.8% of those whose practices remained open. The main difficulties encountered by TCM physicians and therapists during remote consultations were linked to their inability to perform acupuncture (36.6%) and the difficulty in making a TCM diagnosis (26.9%).

The main adaptation made by TCM physicians and therapists to their consultations due to the risk of contamination from COVID-19 was to give up the practice of acupuncture. Indeed, 65.9% (*N =* 211) stated that they did not practice acupuncture from March 16 to April 27.

**Fig. 3:**
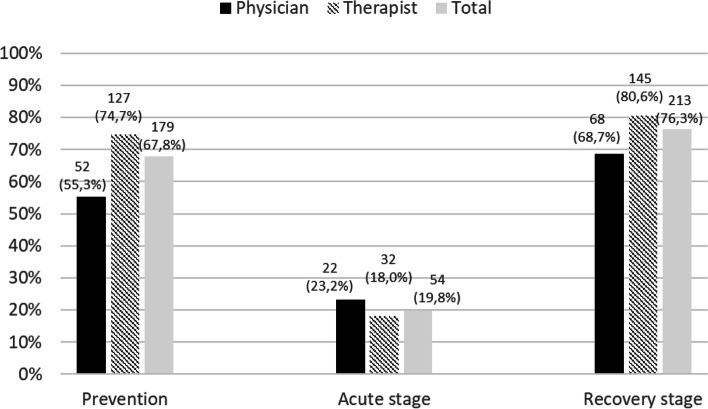
Number and percentage of TCM physicians’ and therapists’ consultations related to the stage of COVID-19 (prevention, acute, recovery)

As part of this outcome, we assessed the proportion of TCM therapists and physicians who consulted at least one patient with respect to COVID-19 from March to September 2020, as well as the prevalence of consultations according to the stage of the disease (prevention, acute, recovery) and its associated factors. We found that 76% of participants (*N =* 242) consulted a patient at least once with respect to COVID-19 (76.1% of therapists and 74.5% of physicians). Overall, physicians and therapists consulted more patients during the recovery and prevention stages than during the acute stage of the disease. Figure 3 summarizes the consultations made by these TCM physicians and therapists according to the three above-mentioned stages.

Acupuncture was the most frequently used technique among TCM therapists and physicians consulting for prevention (*N =* 144, 80.4%) and among those consulting for recovery (*N =* 197, 92.5%), whereas Chinese pharmacopeia was the most used by those consulting for the acute stage (*N =* 32, 59.3%). Chinese pharmacopeia was the second most used technique for the TCM physicians who consulted for prevention (*N =* 13, 25%) and recovery (*N =* 16, 23.5%), and moxibustion was the second most for TCM therapists consulting for both of these stages (*N =* 58, 45.7%; *N =* 62, 42.8%).

Among the respondents who practiced Chinese pharmacopeia, only one formula was cited several times for the same stage (*N =* 14, 27.5%). This formula, named Yu Ping Feng San, is composed of three Chinese herbs (*Astragali radix, Atractylodis macrocephalae rhizoma,* and *Saposhnikoviae radix*) and was used in the prevention stage. There was considerable heterogeneity in the use of other formulas in all stages of the disease. Apart from this formula, the others were cited mostly only once and in rare cases up to four times for the same stage.

## Discussion

Our results provide insights into the influence that legal restrictions linked to the COVID-19 pandemic had on TCM physicians’ and therapists’ practices, as well as on the proportion of respondents who consulted patients with respect to COVID-19.

TCM therapists (73%) closed their practices significantly more than TCM physicians (35%) did during Spring 2020. Indeed, a law restricted the opening of most TCM therapist’ practices from March 16 to April 27, 2020, whereas TCM physicians did not have to close down. Among those who closed their practices during this first period, 25% of TCM physicians and 47.5% of TCM therapists did not consult at all. A study on the impact of COVID-19 on CAM providers in Norway showed similar results with 61% of them not working during the lockdown [24]. Although, we did not assess the financial impact of the legal restrictions on our respondents, it is likely that financial losses were consequent, especially for TCM therapists as most of them had to close their practice. Indeed, a study conducted among general practitioners, also in Switzerland, showed that 99% reported financial losses, although most of their practices remained open [10]. In addition, the closure of practices raises the question of continuity of care among chronically ill patients, as, in Switzerland, this population uses TCM twice as often as the rest of the population [13].

The remaining proportion of TCM physicians and therapists kept on consulting during this first period of Spring 2020 and had to adapt to accept consultations based only on an emergency need, as other healthcare professionals in the country did during this period, for example, general practitioners, osteopaths, occupational therapists, and midwives [6–8, 10]. As in our study, these healthcare professionals predominantly used distant consultations to consult during the semi-lockdown; the main method of communication for most of these professionals in Spring 2020 in Switzerland being the telephone, as well as emails and videoconference [6–8, 10]. The use of distant consultations has been observed worldwide among healthcare providers during the pandemic [26–28], including among CAM providers [24, 25]. Home visits were also often conducted by our respondents even if the practice was open. In our study, the target group of this service was not identified; nevertheless, it is likely that the aim was to protect patients fearing to get infected during transport or in the clinic, as two qualitative studies conducted in Switzerland and Denmark have highlighted [29, 30]. According to a survey carried out among occupational therapists and midwives in Switzerland, the provision of healthcare at a distance cannot substitute for conventional therapy, as touch is an essential part of the work [8]. This was also highlighted in our study, as the main difficulty encountered by TCM physicians and therapists was the inability to practice acupuncture.

More than three quarters of physicians and therapists consulted at least one patient with respect to COVID-19 during the first wave of COVID-19. Among the participants, the most frequently used technique in daily practice was acupuncture. Thus, it is no surprise that it was also the most used technique in managing patients with COVID-19, although a large majority of TCM physicians and therapists stated that, because of the risk of contamination, they reduced the use of this technique from March 16 to April 27. In general, TCM physicians seemed to use a smaller variety of techniques than TCM therapists did. These data are consistent with their TCM course, which is mainly focused on acupuncture and Chinese pharmacopeia [31].

TCM therapists and physicians consulted more during the recovery stage than during the other two stages. Recovery stage was also the one for which nearly all respondents agreed that TCM had a role to play, whereas results were more contrasted for the two other stages. Recovery represents a challenge for the healthcare system [32]. The number of COVID-19 patients who still have symptoms several weeks after their acute infection, called long COVID, varies widely between sources [32, 33]. In Switzerland, according to a cohort study, 26% of the participants had long COVID [34]. As COVID-19 has affected millions of people, long COVID will affect a large number of people who will need medical support. Therefore, it is all the more necessary to scientifically evaluate the therapeutic usefulness of TCM for this purpose and to disseminate the results to health professionals.

TCM is based on syndrome differentiation and provides a specific treatment for each patient [35]. Considering the personalization of treatments, Chinese herbal formulations will differ between patients. This explains the diversity of responses received in relation to the use of Chinese herbs for a given period of COVID-19 treatment. However, the most cited formula in our study, Yu Ping Feng San, used in the prevention stage, is commonly used in prevention programs against COVID-19 in China [18].

### Limitations

One limitation of the study was the low response rate of 15%, which may impair the generalizability of the findings. This could be explained by lack of time of healthcare professionals due to the pandemic, as well as the lower likelihood of TCM therapists and physicians than other healthcare professionals to participate in scientific studies. Although the sample of physicians is representative of the initial population, there may have been a selection bias in therapists, as male therapists were less represented compared with the initial population of men (22.1% vs 35.2%, respectively), as was the age group between 50 and 99 years old compared with the initial population (43.6% vs 57.7%, respectively). The age difference may be because therapists had to complete the questionnaire online, which may have discouraged older people who are less comfortable with computers. Finally, as the questionnaire was sent 1 year after the period analyzed in this study, a recall bias cannot be excluded.

## Conclusion

It is surprising that countries, like Switzerland, did not take the opportunity at the beginning of the pandemic to include TCM in the management of patients suffering from symptoms related to COVID-19 as no treatment from the conventional medicine was available and as China had already developed specific protocols to use it in this context. In face of a pandemic of this magnitude, it is all the more necessary to use the full capacity of the healthcare system.

The restriction concerning the opening of practices in Switzerland induced a loss of the health workforce, especially among TCM therapists. Nonetheless, the results of this study show that TCM therapists, even if the law forced most of them to close their practice, looked for the best way to continue working during this period. This study shows that TCM therapists and physicians consulted patients with respect to COVID-19, especially during the recovery stage. As China seems to have experienced an added value in the use of TCM in the fight against COVID-19, future studies are needed to assess the efficacy and safety of TCM against COVID-19. As there was an agreement among physicians and therapists that TCM is particularly useful in the recovery stage, clinical studies of TCM use in this stage would be of particular interest, especially when considering the prevalence of long COVID and the absence of effective treatment at this time.

## Data Availability

The datasets generated and/or analyzed during the current study are not publicly available due to privacy or ethical restrictions but are available from the corresponding author on reasonable request.

## Abbreviations

ASCA: Swiss Foundation for Complementary Medicine
CAM: Complementary and alternative medicine
TCM: Traditional Chinese medicine
